# Supplementary figures and images for: The Use of Cerebellar Hypoperfusion Assessment in the Differential Diagnosis of Multiple System Atrophy with Parkinsonism and Progressive Supranuclear Palsy-Parkinsonism Predominant
Source: Diagnostics (Basel). 2022 Dec 2;12(12):3022. doi: 10.3390/diagnostics12123022 (PMC9776891; doi:10.3390/diagnostics12123022)

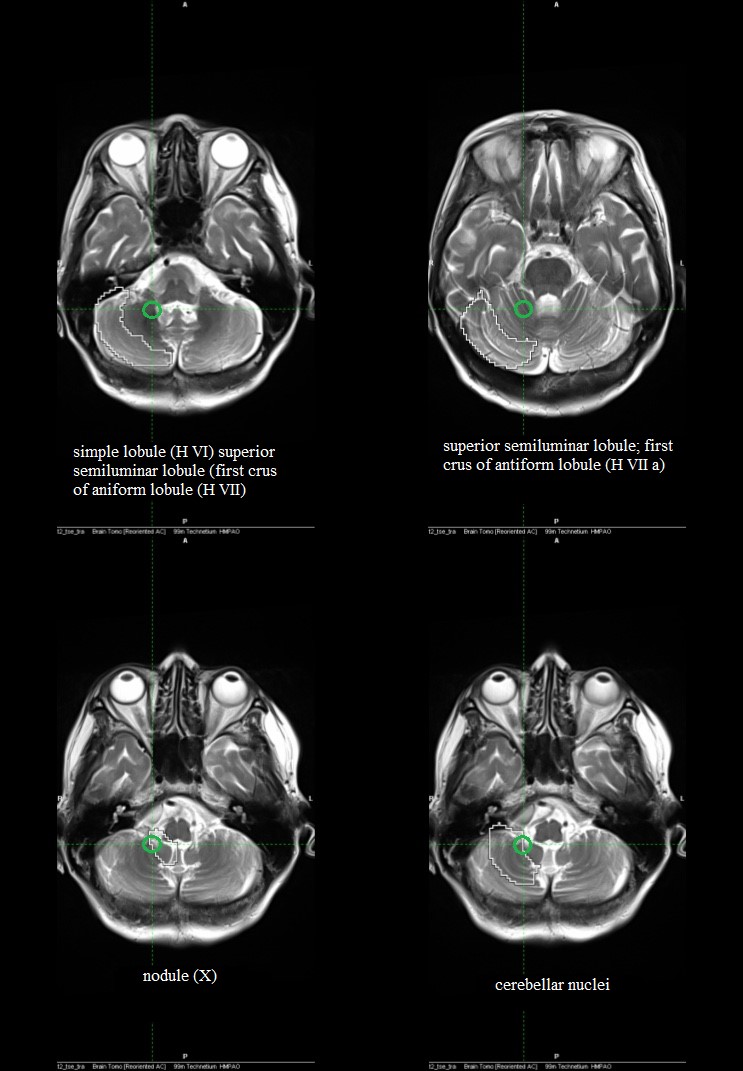

Supplement: Supplementary file 1 [file diagnostics-12-03022-s001.zip › Figure S1. Analyzed ROIs.jpg]

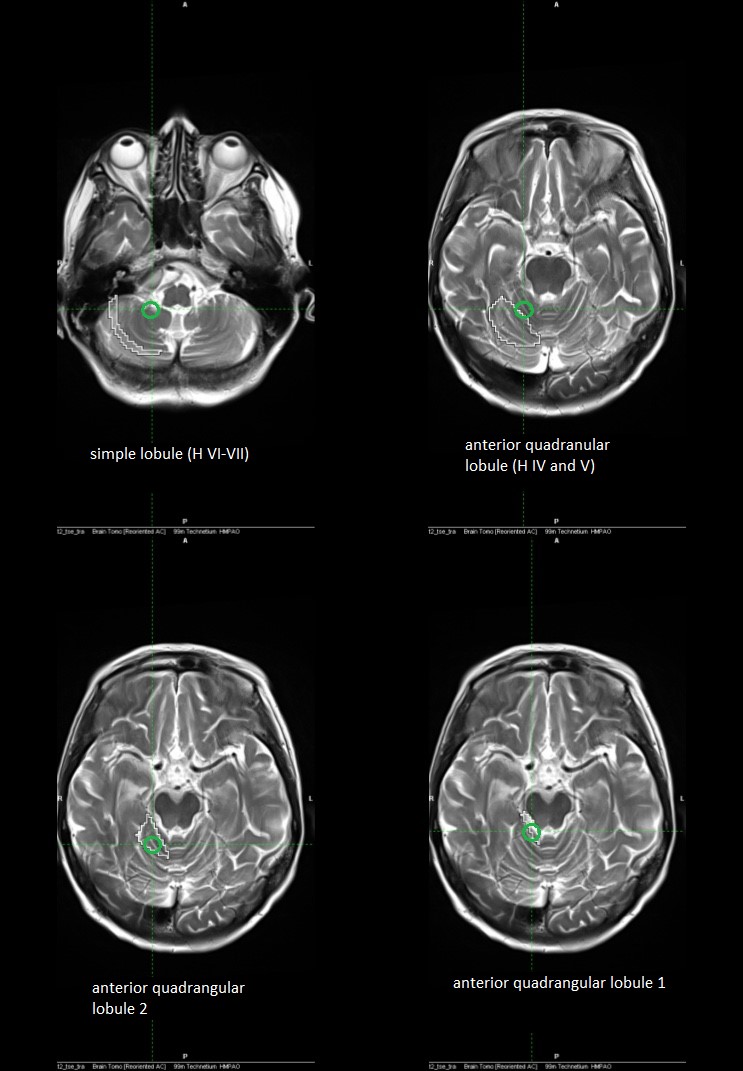

Supplement: Supplementary file 1 [file diagnostics-12-03022-s001.zip › Figure S2. Analyzed ROIs cont..jpg]

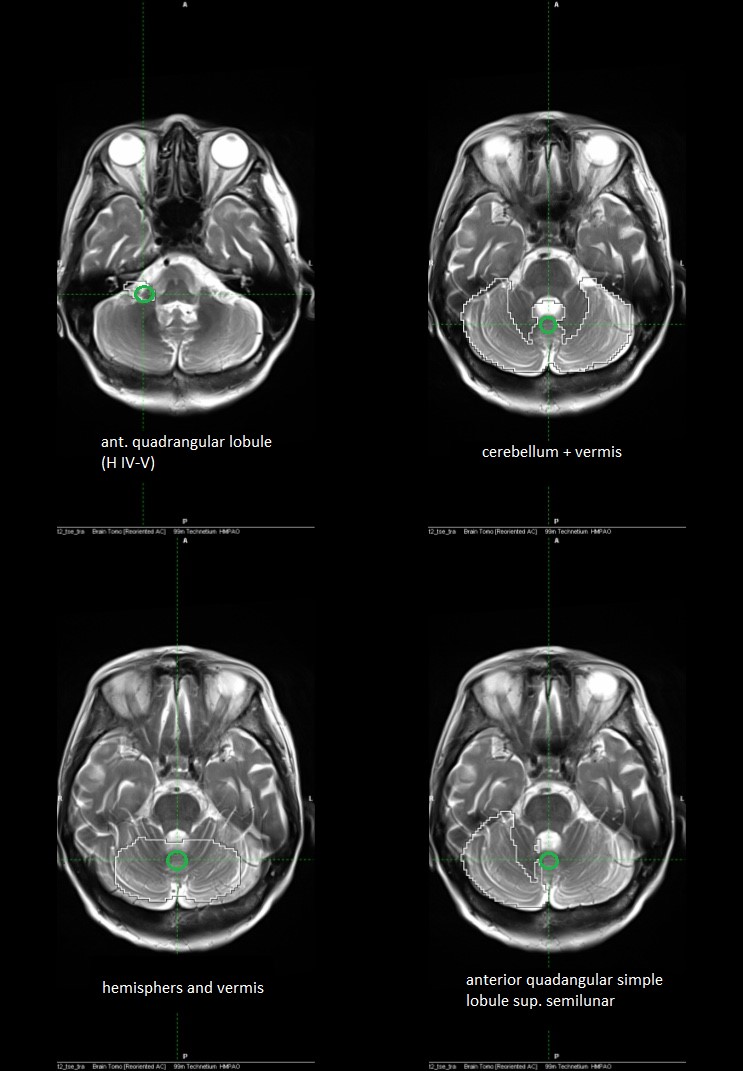

Supplement: Supplementary file 1 [file diagnostics-12-03022-s001.zip › Figure S3. Analyzed ROIs cont..jpg]

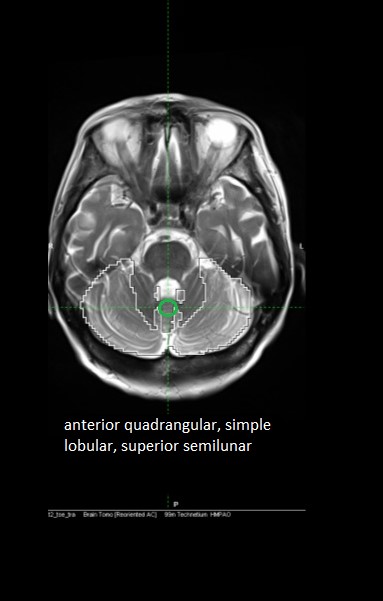

Supplement: Supplementary file 1 [file diagnostics-12-03022-s001.zip › Figure S4. Analyzed ROIs cont..jpg]
